# Patients on outpatient commitment orders in Northern Norway

**DOI:** 10.1186/s12888-017-1331-1

**Published:** 2017-05-02

**Authors:** Henriette Riley, Bjørn Straume, Georg Høyer

**Affiliations:** 1 Department of Community Medicine, Faculty of Health Sciences, UiT The Arctic University of Norway, Tromsø, Norway; 2 Division of Mental Health and Substance Abuse, University Hospital of North Norway, N-9291 Tromsø, Norway

**Keywords:** Coercion, Community treatment order., Outpatient commitment., Community psychiatry.

## Abstract

**Background:**

In recent years, an increasing number of countries have introduced outpatient commitment orders (OC), which imply that patients can be subject to compulsory follow-up and treatment while living in the community. However, few studies on how OC is practised have been published.

**Method:**

Retrospective case register study based on medical files of all patients receiving an OC order in 2008–2012. We used a pre/post design, recording the use of inpatient services three years before and three years after for those patients who received their first ever OC order in 2008 and 2009.

**Results:**

A total of 345 OC orders applying to 286 persons were identified in the study period 2008–2012. Incidence and prevalence rates were relatively stable, but decreased during the last years of the study period. For all the 54 patients receiving their first ever OC order in 2008 and 2009, need for treatment was the reason for imposing OC, and all received psychotropic medication. The number of inpatient admissions and inpatient days was greater, while the number of days for each admission was lower three years after the OC order than three years before. The first ever OC lasted under a year for 76% of the patients. Receiving depot medication and follow-up by psychiatrists predicted longer OC durations than such treatment and care by psychologists. Only nine patients were not hospitalized during the three-year follow-up after the first ever OC order.

**Conclusion:**

Patients on first ever OC orders in Northern Norway used inpatient services more after OC orders than before. Further studies are needed to explore whether increased use of inpatient services by OC patients is beneficial or a failure of OC.

## Background

In recent decades, the majority of jurisdictions in North America, Australia and Europe have introduced legal measures that can compel patients to comply with outpatient treatment [1–3]. The coercive powers of outpatient commitment (OC) vary between jurisdictions, as do the procedures and services offered to patients under an OC order [4, 5]. A variety of names, such as community treatment orders, conditional release, preventive commitment, involuntary outpatient treatment, aggressive or assisted community treatment have been applied to OC in the literature. In this paper, we use the term OC to cover all forms of involuntary outpatient orders.

Norway introduced OC in 1961, as one of the first countries in Europe. The other Scandinavian countries, Sweden and Denmark, first sanctioned OC in 2008 and 2010 respectively [6, 7]. In spite of the long use of OC in Norway, the scheme has never been assessed. The present paper presents results from one of the five sites participating in the larger Norwegian Outpatient Commitment Study, exploring the implementation of OC orders in different catchment areas.

### OC in Norway

According to the earlier Norwegian 1961 Mental Health Act, patients discharged from inpatient care with an OC were obliged to attend their treatment appointments, if necessary by use of force. In addition, readmissions to inpatient care were facilitated for patients on an OC order, as no new independent assessment of the patient was needed for readmission. If a patient on an OC order refused to comply with the treatment, the law required a separate order for compulsory treatment. The Norwegian Mental Health Act was revised in 1999 and 2006, but few fundamental changes were made concerning OC, except for the introduction of the possibility to issue an OC order without a prior period as an inpatient, an option that is rarely used.

The criteria for OC in Norway are the same as those required for patients subject to inpatient involuntary psychiatric care. The decision to place a patient on an OC order is valid for one year, and only authorized psychiatrists or clinical psychologists employed by the specialized mental health services can make such decisions. A special feature of the legal framework governing OC in Norway is that the order continues to run during inpatient periods taking place within the valid period of the OC order. OC orders may be renewed for one year at a time by application to an independent review board (the Control Commission). There is no limit to the number of times the order may be renewed. In addition, the patient must be assessed every three months, by either a psychiatrist or a psychologist authorized to make OC decisions, to determine whether the legal criteria for OC are still fulfilled; if not, the order must immediately be lifted.

Patients and their next of kin can appeal the decision to place the patient under an OC order to the independent Control Commission, and the decision of the Control Commission can, in turn, be further appealed in court. OC legislation also requires a treatment plan to be drawn up, unless the patient refuses such a plan.

According to the latest available governmental mental health statistics, there were 2364 patients on an OC order in 2013 [8], corresponding to an OC prevalence rate of 61.1 per 100,000 inhabitants aged 18 years and above. These figures should be read with caution, as there are concerns about the quality of OC statistics in Norway [8].

### Aim of the study

The aim of the study was threefold: Firstly, we wanted to produce reliable incidence and prevalence data on OC in the catchment area. Secondly, we aimed to explore how OC is practised in a region in Northern Norway, and thirdly, we wished to determine whether OC had an impact on the use of inpatient services.

## Methods

The study was a retrospective case register study combined with an uncontrolled before/after design for the consumption of health care services by patients receiving OC orders in the two northernmost counties in Norway (Troms and Finnmark). The catchment area had a total population of 232,437 on 1 January 2012, of which 180,394 were aged 18 years or above, and covers approximately 71,000 sq. km (27,413 sq. miles). By comparison, the whole of Denmark is 43,000 sq. km (16,500 sq. miles). There is only one mental hospital serving the two counties, and it is also the only institution authorized to make OC decisions. The catchment area is generally scarcely populated with only three towns of more than 10,000 inhabitants. Many patients have to travel long distances, up to nearly 1000 km by road, and travel is often difficult due to harsh weather conditions.

All patients on an OC order at the beginning of 2008 and living in the catchment area were included in the study. Further, all new OC orders made from 1 January 2008 until 31 December 2012 were added to the study file. We identified 286 patients, representing 345 OC orders, during the period. Among those 286 patients, we further identified all patients with a first ever OC order (index OC) from 1 January 2008 to 31 December 2009 (*n* = 54). For those patients we gathered more comprehensive data, including their consumption of mental health inpatient care three years before and after the first ever OC decision was made. All data were recorded based on information in the patients’ electronic medical files. We searched all files in all mental health care institutions in the study catchment area where OC orders potentially could be made, but found that OC orders were only made by authorized staff at the only psychiatric hospital serving the two counties included in the study. To identify those with an OC order we searched summary reports of all patients discharged from inpatient care. The data extracted from the patient files were recorded onto a specially prepared registration form. Two different persons proofread all data once, while all variables on the use of inpatient services, forced medication and complaints were proofread twice. In addition, a random sample of case files was proofread for all variables for a third time. As no errors were identified in the random sample, we did not proceed to a third proofreading of all records. Patients that did not live in the catchment area and those who received an OC order issued solely for the purpose of a short-term stay in e.g. a somatic hospital or for transport to other institutions were excluded (*n* = 10).

### Analysis

The data were analysed using the SPSS statistical package 22. The statistical analysis included the chi-square test, the T-test, and one sample Kolmogorov-Smirnov non-parametric test. Significance levels are indicated by asterisks in the tables. The main analysis was based on survival analysis methods with time starting at first OC decision and end of first OC period as the censoring event. Follow-up of those with a first ever OC order took place until 31 December 2012.

The following variables were evaluated as possible factors influencing the duration of OC by Kaplan-Meier analysis and log-rank tests: sex, age, diagnosis, time since first contact with psychiatric services, use of psychiatric services before OC, living alone or not, place of residence, involuntary admissions before first ever OC order, reference to danger to self or others, treatment compliance, medication (forced, voluntary and depot), and follow-up variables during OC. We first made a bivariate analysis entering all variables that could have any possible influence on the duration of the OC order, and then entered all variables with high correlations as independent variables in the final multiple regression analysis. The independent variables included in the final Cox regression model are accounted for in the results section.

### Ethical considerations

The study was approved by the Regional Committee for Medical and Health Research Ethics, Region North (REC North) (Project No. 2010/2268), and conducted in accordance with the Declaration of Helsinki. To be able to produce reliable data on incidence and prevalence of OC in the study catchment area, completeness of the data was crucial. For this reason, REC North granted access to the medical files, without obtaining individual consent by the patients. All data were de-identified before being stored and used in the analysis.

## Results

### Incidence and prevalence of OC

The incidence of OC orders in the years 2008–2012 varied between 40 and 60 new orders per year, corresponding to an incidence rate of between 22.1 and 33.2 per 100,000 population 18 years or above (Table 1). On average, 8.2% of all involuntary inpatient admissions continued as OC over the five study years. Point prevalence rates on 1 January, for the same years, varied between 59.8 and 72.1 per 100,000 population 18 years or above. After an initial increase in incidence and prevalence rates, there was a tendency for both rates to decrease in the final years covered by the study. All first ever OC orders took effect on discharge from an inpatient period. Four patients received their first ever OC in conjunction with their first ever inpatient admission.

**Table 1:** Incidence and prevalence of OC in absolute numbers and rates, and proportion (%) of OC patients of all discharged involuntarily admitted patients as of January 1st 2008–2013 in Northern Norway. Rates are per 100,000 population above 18 years of age. Based on 345 OC orders for 286 patients in the period 2008–2012

	2008	2009	2010	2011	2012
Incidence					
New OC orders, absolute numbers	57	60	57	49	40
Incidence rates	31.5	33.2	31.5	27.1	22.1
Number of involuntary inpatient admissions	608	643	695	692	553
Percentage discharged with an OC order	9.4	9.3	8.2	7.1	7.2
Prevalence					
Point prevalence; number of persons	108	118	130	123	109
Point prevalence rates	59.8	65.4	72.1	68.1	60.4

### Who are the patients on a first ever OC order?

The 54 patients subject to their first ever OC order were predominantly males (68%). The males were significantly younger than their female counterparts, with a mean age of 44 for males and 53 for females (*p* < 0.01). All but 15 patients had a main diagnosis in the schizophrenia spectrum (F20–29, ICD-10), and fourteen (all males) had a comorbid substance abuse disorder (Table 2). There was no significant difference in age or gender between those on a first ever OC order in 2008–2009 (*n* = 54) and other patients with an OC order included in the study (*n* = 232). Thirty-seven (70.6%) of patients with a first ever OC order lived in a private flat or house, of whom 21 (39.4%) lived with their family, a spouse or a friend. However, the majority of the patients (59.6%, *n* = 32) lived alone, and 75.5% (*n* = 40) had their only income from the national insurance scheme or received social benefits, while 11.3% (*n* = 6) of the patients had no income at all. Thirty-eight (74.5%) of the patients with an first ever OC order had a history as inpatients for three years or more prior to receiving this order, but males had a significantly shorter history as inpatients than females (*p* < 0.05).

**Table 2:** Characteristics of patients who received their first ever OC order in 2008 and 2009 in Northern Norway by gender (*n* = 54)

		Gender*	
	Female	Male	Total
	*n* = 18	*n* = 36	*n* = 54
Age**			
18–39	1	15	16
40–87	17	21	38
Mean**	52.7	44.4	47.1
Range	29–84	21–87	21–87
Lives with:			
Spouse/partner	4	1	5
Family	7	9	16
Alone	7	21	28
Homeless/Other	0	3	3
Accommodation:			
Private house/flat	15	22	37
Supervised council housing	1	4	5
Staffed council housing	1	4	5
Homeless/Other	1	3	5
Income			
Salary	1	6	7
Insurance/social benefits	15	25	40
No income	2	4	6
Diagnosis			
F20–29 (Schizophrenia spectrum)	13	26	39
F31, F33 (Bipolar and depression)	4	7	11
Other (F06, F19)	1	3	4
Comorbid substance abuse **			
Yes	0	14	14
No	18	22	40
Time since first inpatient episode (3 missing)*			
< 3 years	1	12	13
> 3 years	16	22	38

*p < 0.05, **p < 0.01

### Justification for the OC order and treatment provided to OC patients

Psychiatrists made almost all the first ever OC orders in this study. Authorized psychologists only made two such primary decisions. The number of psychologists responsible for OC patients increased to five during follow-up of the OC order of the included patients. When justifying the OC decision, decision makers referred to the need for treatment in all cases, except for one case where the information was missing (Table 3). In all but one case, decision makers specified the need for treatment as a need for medication. Seven patients were said to also represent a danger to themselves or others. Dangerousness alone was not a justification in any cases. In ten cases, decision makers discussed the feasibility of voluntary care, but concluded that voluntary care was unrealistic. The impact of substance abuse as an additional reason for imposing an OC was only raised in eight cases.

**Table 3:** Justification of OC orders as they appear in patients’ records. All decisions on first ever OC in Northern Norway in the years 2008 and 2009 by gender (*n* = 54)

		Gender	
	Female	Male	Total
Need for treatment			
Discussed and confirmed^a^	17	36	53
Discussed, not confirmed	0	0	0
Not mentioned	1	0	1
Danger to self			
Discussed and confirmed	2	2	4
Discussed, not confirmed	2	5	7
Not mentioned	14	29	43
Danger to others			
Discussed and confirmed	0	3	3
Discussed, not confirmed	3	2	5
Not mentioned	15	31	46
Voluntary care unrealistic			
Discussed and confirmed	5	5	10
Discussed, not confirmed	0	2	2
Not mentioned	13	29	42
Substance abuse			
Discussed and confirmed	0	8	8
Discussed, not confirmed	6	10	16
Not mentioned	12	18	30

^a^“Discussed” means that the issue was discussed in the medical file, while “confirmed” means that the issue was explicitly mentioned in the OC decision

Individual treatment plans were drawn up for 20 of the patients, while five patients objected to such plans being made. The remaining 29 patients lacked a treatment plan. All patients on OC received psychotropic drugs, seven of them for only parts of the OC period. Twenty-one patients (38.9%) were on depot medication, and 31 patients (57.4%) had one or more parallel decisions authorizing involuntary medication, for either part or the whole of the OC period. Forty-six percent of the patients had regular appointments with health care professionals more frequently than every second week. When those who saw treatment staff every second week were added, the percentage rose to 70%. Other treatment modalities than pharmaceutical treatment were mentioned in the patient records for 39 patients.

### Duration of first ever OC orders

Median duration of the first OC order was 161 days for females and 211 days for males, and mean duration was 370 days for both genders (Table 4). Seventy-six percent of the patients had their OC terminated less than a year after the order was made, and for 76% of those patients the order was lifted by a formal decision made by authorized OC decision makers. In eight cases, we found no formal decision to end the OC, but as no applications to renew the order were filed, the OC terminated when the primary OC decision expired. Five patients were still on their first ever OC order three years after the order was implemented. We found that those with a first ever OC order in 2008 and 2009 (*n* = 54) had a significantly shorter OC period than those with a new, but not the first, OC order in the same years (*n* = 45) (*p* < 0.01). Being on depot medication and being followed up by psychiatrists compared to psychologists predicted longer durations of OC (Cox regression analysis, *p* < 0.01 for both predictors).

**Table 4:** Duration of first ever OC orders. All patients on first ever OC order in Northern Norway in the years 2008 and 2009 by gender (*N* = 54)

		Gender	
	Female	Male	Total
Mean duration (days)	370	370	370
Range (days)	18–1795	8–1488	8–1795
Median (days)	161	211	202
IQR	71–354	147,5–352,5	127–354
Less than 1 year	14	27	41
1–2 years	1	5	6
More than 2 years	3	4	7

### Use of inpatient care three years before and three years after the first OC order

Patients who received their first ever OC order had significantly more inpatient episodes and a greater mean total number of inpatient days in the three years after the order compared to the three years before (*p* < 0.01) (Table 5). The mean duration of stay per admission decreased from 26 days before the OC order to 15 days after (*p* < 0.01). Time to first inpatient readmission episode after the first ever OC order varied between three and 1016 days, with mean and median duration of 145 and 38 days respectively. No significant predictors for the duration between the first ever OC order and first readmission to a psychiatric facility were found using age, gender, forced treatment order, substance abuse, time since first inpatient episode, and number of compulsory admissions three years before index OC as potential explanatory variables in a Cox regression analysis. Neither did we find that those with short or long inpatient stays before the OC order had similar patterns of inpatient stays three years after the order. Nine of the patients with a first ever OC order were not readmitted during the three-year follow-up period, but only one of those was included in the analysis, as the eight others had their first contact with the mental health services fewer than three years before the OC order was made.

**Table 5:** Use of inpatient care for patients on their first ever OC order, three years before and three years after their index OC (*n* = 38)^a^

	Three years before Index OC	Three years after Index OC
Number of inpatient admissions *		
Mean	3	7.3
Median	2	3
Range	1–14	0-48^b^
Total number of inpatient days*		
Mean	77.1	109.5
Median	48	60.5
Range	7–174	0-717^b^
Mean duration of inpatient stay per admission*	25.7	15

* *p* < 0.01

^a^16 patients with less than three years observation time as mental health care patients before the index OC were excluded from the analysis

^b^One person had no admissions three years after the index OC

## Discussion

### Patient characteristics and OC incidence and prevalence rates

The patients in this study revealed the same socio-demographic characteristics as those found in most studies of OC patients [1, 9–12]. The typical OC patient seems to be a middle-aged male with a diagnosis in the schizophrenia spectrum, living alone or with his family, with no income, except from public insurance or social benefits. It is interesting that in spite of different legislation and different traditions in various countries, it is the same group of patients that seem to be subject to OC. Both incidence and prevalence rates of OC increased over the first two to three years of the study period, after which both rates decreased. This was a somewhat surprising finding, as the general impression is that OC has been constantly increasing in recent years, both in Norway and internationally [8, 11, 13, 14]. Unfortunately, as there are no national statistics in Norway reporting OC incidence and prevalence rates on a regular basis, it is impossible to compare the rates found in Northern Norway to national statistics. The only available figure for comparison is the national one-year prevalence rate for 2013 of 61.1 per 100,000 population 18 years or older [8]. This figure corresponds well with the prevalence rate we found in our study in 2012. We are confident that we managed to capture all OC decisions made during the study period as we checked the files at all sites in the catchment area that could possibly have made OC decisions, and because we were allowed to access files without the consent of the patient. In addition, the computer system used to manage patients’ files has a default setting that makes it impossible to discharge a patient from inpatient care without selecting whether the patient is subject to OC at discharge or not. The number of both acute and other hospital beds in psychiatric facilities remained stable during the study years, indicating that the capacity of inpatient services was unlikely to have had an impact on the OC incidence rates found in this study.

### Justification for making an OC order

For patients receiving a first ever OC order, need for treatment was the main reason for imposing the order. This finding was expected, given that the target group for OC comprises those who need follow-up and tend to drop out of treatment once discharged from inpatient care [4, 15, 16]. Only seven patients were said to be dangerous. This is also an expected finding, as clinicians would be reluctant to discharge patients who pose a danger to either themselves or others. Nevertheless, it is somewhat surprising that decision makers did not discuss or even mention dangerousness at all, except in those seven cases. Likewise, the feasibility of voluntary care was not considered or mentioned in 78% of the OC decisions, despite the legal requirement of the Norwegian Mental Health Act to establish that no voluntary alternatives have worked, or appear to be realistic, before outpatient commitment can be imposed. The general legal principle of proportionality, and then to always use the least restrictive alternative, should in itself encourage clinicians to address the issue of voluntary alternatives, before a decision on involuntary care is taken.

In a larger context, the issue of how coercive intervention in mental health care is justified touches upon the debate on which criteria can be used to authorize coercive care within a legal and human rights oriented framework. Over recent decades, an increasing number of jurisdictions worldwide have restricted civil commitment of patients with mental disorders to apply only to patients who are dangerous to themselves or others [17]. While most countries in Europe to date have excluded the need for treatment as a criterion for civil commitment, the treatment criterion has survived in all Scandinavian countries [6, 7, 18]. Against this background, two questions arise. Firstly, whether OC can be justified at all in jurisdictions where the need for treatment is no longer a ground for civil commitment. Secondly, whether dangerousness is under-communicated in jurisdictions that still use the need for treatment criterion, as clinicians find it less stigmatizing to refer to treatment needs than dangerousness. We are not aware of any studies or publications that can substantiate or disprove the reality of this argument. However, it may have a bearing on our finding that clinicians used the need for treatment criterion as justification for all patients with their first ever OC, and rarely discussed dangerousness.

### Treatment provided to OC patients

Psychopharmacological treatment was the dominating element in the treatment during the OC period. All patients received psychotropic medication, in practice synonymous with neuroleptics, which were depot injections in 21 cases. In Norway, involuntary hospitalization or OC orders do not in themselves authorize any kind of treatment without the consent of the patient. If a civilly committed inpatient or an OC patient refuses treatment, a separate decision on forced treatment must be issued. Just over half of the patients (57%) had a parallel decision on forced treatment, implying that almost half adhered to the drug treatment on a voluntary basis. It can be questioned to what degree patients on an OC order are able to give free and valid consent to drug treatment, or whether they take the medication voluntarily. The preamble to a planned revision of the Norwegian Mental Health Act from 2011 argues as follows: “Because civil commitment is a necessary precaution for forced treatment, it is most likely that the great majority of OC patients either have a forced medication order or comply with medication because they consider that if they do not, they will receive a forced medication order” (15 p. 93, our translation). Similar findings are reported in qualitative studies from the Norwegian OC study [19, 20].

The descriptions in the patients’ files of their needs for other treatment modalities than medication were vague. The kind of treatment mentioned varied greatly, from psychotherapy to minor structural issues concerning the patients’ everyday life, and it was difficult to assess the real content of treatment other than medication. Qualitative interviews with patients on OC and their relatives reveal that the content of the treatment was almost exclusively centred on medication [19–21]. We are not aware of other studies on OC that have detailed the content of various therapeutic activities offered to OC patients in different countries.

### Duration of first ever OC

The majority of first ever OC orders were lifted within one year, with a median duration of less than half a year for women and about seven months for men. This is the only study to date that presents data on the duration of OC orders in Norway, and the duration found here can therefore not be compared with national practice. In reports from other countries, Burns and colleagues [22] found that the median duration of all OC orders during the three-year study period was 364 days. Smith et al. [23] found that 51% of the index OCs lasted for more than one year, while Lera-Calatayud et al. [12] found a mean duration of 29.2 months (standard deviation 16.5) in their study.

Being on depot medication and having a psychologist responsible for following up were the only variables that ended up as significant predictors of duration of OC, but neither of them appeared as self-evident or easy to interpret. We first entered all variables that emerged as being, or were close to being, significant in bivariate analysis in the multivariate Cox regression analysis, and then excluded variables that emerged with low impact on the duration of the OC period step by step. Variables we thought had a stronger impact on duration, like having a forced treatment order, co-morbid substance abuse, living alone, distance from patient to hospital and number of previous inpatient episodes, both voluntary and involuntary, did not explain much of the variance. Finally, we ended up with a model entering age, sex, being on depot medication, being followed up by a psychologist or psychiatrist and how long the patient had received specialized mental health services. Regarding the 39 % of the patients receiving depot medication, we could not find any other differences between those receiving depot and oral medication. We suspect that being on depot medication can be a confounder and that our data have not captured traits or qualities among patients receiving depot medication that are more valid. Other studies have shown mixed results; some report reduction in depot use over time for OC patients [2, 24] although others show the opposite effect [25, 26]. The difference between psychologists and psychiatrists in relation to duration of the OC period must also be interpreted with caution. The low number of psychologists could represent a selection bias related to attitudes to coercive treatment that are not representative of psychologists in general, and likewise the patients under their responsibility could be selected as well. Larger scale studies are needed to establish whether there are real differences between psychologists and psychiatrists in OC practice.

### Use of inpatient care before and after the first ever OC episode

The finding that use of inpatient care increased after the first ever OC episode is noteworthy. Given the ideology underpinning the use of OC in Norway, the order should in principle serve as a less restrictive alternative to inpatient care [1, 15]. On this basis, we had expected a decrease in inpatient days after the implementation of an OC order. Internationally, the effect of OC on the use of health services has been extensively studied, with conflicting results [3, 16, 27]. Review papers, including one Cochrane review [1, 16, 28, 29], all report that the three RCTs on OC published so far [9, 30, 31] found no effect on the use of health care services, neither did a meta-analysis of the same three RCTs [27, 32]. Non-randomized controlled and uncontrolled studies show mixed results regarding readmission rates and hospital days before and after an OC order [2, 33]. A possible contribution to the increase in inpatient admissions and inpatient days found in the present study after an OC order may be the extremely long distances between the psychiatric hospital responsible for patients on OC and some OC patients (up to nearly 1000 km or 620 miles). The long distances in the study catchment area often require OC patients to be hospitalized when undergoing the mandatory assessments of OC patients by hospital staff on a three-monthly basis as required by law. We further tried to record how many of the admissions after the OC order came into force were voluntary or involuntary, but poor quality of the data in the patients’ files made it impossible to produce reliable data on this issue.

### General comments on the use of OC

The debate on OC has often raised the question of whether OC works or not. So far, there has been no clear answer to this question. One problem in this context is that the aims of OC orders are not well defined, and vary among jurisdictions. The legal criteria for OC will also vary according to the aims in different jurisdictions. Furthermore, there are indications of variations in how OC is practised within individual jurisdictions [1, 4]. Given this state of affairs, effect measures will remain equally ambiguous and difficult to apply across jurisdictions. Readmission to inpatient care has been by far the most commonly used variable in effect studies on OC. However, the validity of readmissions as an outcome measure is rarely discussed. It appears to be more or less taken for granted that readmission of OC patients is always a sign of failure. Findings from qualitative studies have showed that patients on OC appreciate being able to get inpatient care as long as it is on their own initiative and terms [20]. In our view, readmissions on a voluntary basis attending to needs as expressed by patients could rather be considered a success criterion of the scheme, in contrast to involuntary, unplanned and unwanted readmissions. Few, if any, studies on the effect of OC have made this distinction. It can also be questioned whether legal frameworks of different OC schemes, combined with available resources and practical matters related to OC implementation, may in themselves generate the unintended use of inpatient care for patients on OC orders. The long journeys for many patients included in this study may be an example of such factors. The short follow-up period in most OC studies, usually one year or less, also contributes to uncertainty about OC effects. All the factors mentioned above, and probably others not recognized here, may partly explain why OC studies so far show no effect, or are inconclusive. Given the current situation, we concur with other authors advocating the need for more and better designed studies on OC [2, 10, 22], based on more comprehensive data, more relevant outcome measures and longer follow-up periods.

### Limitations

The catchment area is unusual, with long distances between the patients’ home and hospital, and may be not representative of other catchment areas in Norway. Further, the study has a small sample and there are no reference national data to compare with. Finally, the data collection method was limited to information in the patients’ electronic medical files, and results of this study can only reflect what was recorded by staff.

## Conclusions

Annual incidence and prevalence rates were relatively stable over the five-year period covered by the study (2008–2012), corresponding to an average of 53 new OC decisions a year and 118 persons on OC at a given time each year. OC orders were made in 8 % of all involuntary admissions in the same period. Patients on OC were dominantly middle-aged men with a schizophrenia spectrum diagnosis. Males were significantly younger, and had significantly shorter history as inpatients, than their female counterparts. In all but one case, OC orders were justified by the need for treatment, specified as the need for medication. Dangerousness was mentioned as an additional issue in only seven cases. All patients received psychotropic medication during their OC period, whereof 39% as depot injections. The OC order was lifted within one year for the majority of patients (76%). Being on depot medication and being followed up by psychiatrists compared to psychologists predicted longer durations of OC. Patients on their first time ever OC order had significantly more inpatient episodes and a greater mean total number of inpatient days in the three years after the order compared to the three years before, but the mean duration of stay per admission decreased from 26 days before the OC order to 15 days after. The validity of the use of inpatient services as an outcome measure in studies of OC can be questioned. In particular, the common interpretation of re-hospitalization as a failure of OC should be reconsidered.

## Abbreviations

OC: Outpatient commitment
REC North: Regional Committee for Medical and Health Research Ethics, Region North
